# Abnormal Ventral Somatomotor Network Homogeneity in Patients With Temporal Lobe Epilepsy

**DOI:** 10.3389/fpsyt.2022.877956

**Published:** 2022-06-17

**Authors:** Dongbin Li, Ruoshi Liu, Lili Meng, Pingan Xiong, Hongwei Ren, Liming Zhang, Yujun Gao

**Affiliations:** ^1^Department of Neurology, The First Affiliated Hospital of Harbin Medical University, Harbin, China; ^2^First Department of Neurology and Neuroscience Center, Heilongjiang Provincial Hospital, Harbin, China; ^3^Department of Neurology, The Fourth Affiliated Hospital of Harbin Medical University, Harbin, China; ^4^Department of Psychiatry, Wuhan Mental Health Center, Wuhan, China; ^5^Department of Sleep, Wuhan Hospital for Psychotherapy, Wuhan, China; ^6^Department of Taihe Hospital Reproductive Medicine Center Affiliated To Hubei University of Medicine, Shiyan, China; ^7^Department of Medical Imaging, Tianyou Hospital Affiliated To Wuhan University of Science and Technology, Wuhan, China; ^8^Department of Psychiatry, Renmin Hospital of Wuhan University, Wuhan, China

**Keywords:** temporal lobe epilepsy, ventral somatomotor network, network homogeneity, resting-state functional magnetic resonance imaging, executive function

## Abstract

**Background:**

Abnormalities of functional connectivity in the somatomotor network have been thought to play an essential role in the pathophysiology of epilepsy. However, there has been no network homogeneity (NH) study about the ventral somatomotor network (VSN) in patients with temporal lobe epilepsy (TLE). Therefore, we explored the NH of the VSN in TLE patients in this study.

**Methods:**

The sample included 52 patients with left temporal lobe epilepsy, 83 patients with right temporal lobe epilepsy, and 68 healthy controls. The NH method was utilized to analyze the resting-state functional magnetic resonance imaging data.

**Results:**

Compared to the controls, rTLE patients had significantly higher NH in the bilateral postcentral gyrus, and significantly lower NH in the bilateral Rolandic operculum and the right superior temporal gyrus (STG). The NH values of the left postcentral gyrus were significantly higher in lTLE patients than in the healthy controls, and lTLE patients had lower NH in the right Rolandic operculum. The altered NH in the postcentral gyrus was negatively correlated with the illness duration, and the decreased NH in the left Rolandic operculum was negatively correlated with the executive control reaction time (ECRT).

**Conclusion:**

Our findings suggest that altered NH of the postcentral gyrus, Rolandic operculum and STG might be associated with the pathophysiology of TLE, and thus, highlight the contribution of the VSN to the pathophysiology of TLE.

## Introduction

Epilepsy is one of the most common neurological disorders, affecting over 70 million people worldwide and putting a considerable strain on health-care infrastructure and the economy (1). Temporal lobe epilepsy (TLE) is the most common type of partial epilepsy referred for surgery, accounting for more than 40% of surgical cases, because of the failure of antiepileptic drug therapy (2). TLE is characterized by cognitive dysfunction, which includes memory, executive functioning, and general intellectual functioning disorders, thus contributing to a poor quality of life (3). Although temporal lobectomy results in seizure-free status in 70% of TLE patients (4), surgical resection is still invasive and has undesirable side effects. Recent work has linked TLE with network-level disruption (5), and resting-state functional magnetic resonance imaging (rs-fMRI) studies have revealed its neural connections. MRI is thought to be the most direct method for demonstrating functional connectivity among separated regions of the brain at rest and is the primary method used for studying brain networks.

The somatomotor network is a resting-state network composed of bilateral pre- and postcentral gyri. Based on large amounts of resting-state fMRI data and a data-driven clustering approach (6), Biswal et al. (7) found that the somatomotor network belongs to the conventional group of seven cortical neuronal networks. Several studies have confirmed that the somatomotor network has strong positive connectivity with various brain areas, including the ventral attention networks, frontoparietal networks, and default mode networks (8). In addition, the somatomotor network is closely correlated with the occurrence and progression of diseases such as autism spectrum disorder, schizophrenia, and major depressive disorder (8–10). However, there have been a few reports on the somatomotor network in patients with epilepsy. Furthermore, the symptoms of epilepsy, such as myotonia, myoclonia, and atonic seizures, are closely related to the motor system. In this study, we focused our work on the potential functional mechanism of the ventral somatomotor network (VSN), which is a subdivision of the somatomotor network, in patients with TLE.

With the development of imaging technology in recent years, many brain structural and functional differences have been reported in patients with TLE and healthy controls. For example, Gao et al. (11) found a decrease in functional connectivity and structural deficits in the alerting network of patients with right-sided TLE (rTLE) by using the seed-based functional connectivity method. Mankinen et al. (12) found that interictal epileptiform activity may lead to the reorganization of resting-state brain networks by using independent component analysis (ICA). The seed-based region-of-interest (ROI) functional connectivity method and ICA are two approaches that are often used to assess brain networks. However, these analytical methods have several disadvantages. For example, ROI seed-based methods are critical for selecting an *a priori* ROI within a network. Unlike the ROI seed-based method, ICA does not require an *a priori* definition of seed regions; however, the results are highly dependent on the number of components the algorithm is asked to produce (13). To address these issues, an unbiased method for assessing imaging data is critical.

Network homogeneity (NH) (14) is a survey method that has been widely used in investigating many diseases, such as attention-deficit/hyperactivity disorder and depression, and has also been used to assess connectivity among brain networks (14, 15). The NH method is a novel method that combines the advantages of ICA and ROI seed-based functional connectivity. Thus, it provides an unbiased, hypothesis-driven measure for evaluating a specific brain network associated with a pathophysiologic process or a disorder. The NH method investigates a given network without requiring prior knowledge of the location of network abnormalities. As a voxel-wise survey, NH can be utilized to assess the connectivity of a voxel with the other brain voxels in a predefined network. Homogeneity is defined as the average connectivity of a given voxel. However, VSN homogeneity in patients with TLE has not been reported. In the present study, we analyzed rs-fMRI data from patients with TLE to investigate abnormalities in the NH of the VSN and explore the potential mechanism of impaired somatomotor function in TLE.

## Materials and Approaches

### Subjects

Fifty-two patients with left temporal lobe epilepsy (lTLE) and 83 patients with right temporal lobe epilepsy (rTLE) were recruited from the Department of Neurology, Tianyou Hospital Affiliated to Wuhan University of Science and Technology. Sixty-eight healthy people were recruited from those who underwent a standard physical examination at the medical examination center of the Tianyou Hospital Affiliated to Wuhan University of Science and Technology. Patients were diagnosed with TLE based on the diagnostic manual from the International League Against Epilepsy (16). The inclusion criteria for TLE were as follows (epilepsy patients who met any two of the following symptoms): (1) the clinical onset of symptoms suggested epileptic foci in the temporal lobe, such as psychiatric symptoms, abnormal emotional experiences, automatisms, epigastric rising, or dystonic posturing of the limb; (2) the imaging results showed atrophy or sclerosis in the right/left temporal lobe; and (3) the interictal electroencephalographic traces suggested an abnormality in the right/left temporal lobe. The exclusion criteria for all subjects were as follows: (1) left-handed; (2) any lifetime psychiatric disorder; (3) history of serious medical diseases or other neurological illness; and (4) Mini-Mental State Examination scores (MMSE) < 24. All subjects gave written, informed consent before participating in the study. All participants were right-handed, and the groups were matched by age, education level, and sex ratio. Our study was performed according to the Declaration of Helsinki and approved by the Medical Ethics Committee of the Tianyou Hospital Affiliated to Wuhan University of Science and Technology.

### Behavioral Paradigm

The executive function was assessed by the attentional network test (ANT) (17). The stimulus signals of ANT visually appear on a screen, and the subjects were required to correctly and quickly identify the orientation in which a central target arrow pointed. The reaction time (RT) of all the subjects were recorded, and the executive control reaction time (ECRT) was calculated by subtracting the consistent arrow direction RT from the inconsistent arrow direction RT (17). A longer ECRT indicated inferior executive control performance.

### Scan Acquisition

Images were acquired with an Achieva 3T MRI scanner (Philips, Amsterdam, the Netherlands) for resting-state functional magnetic resonance imaging (rsfMRI). “Participants were instructed to lie down with their eyes closed and remain awake. A prototype quadrature birdcage head coil filled with foam padding was used to limit the head motion. The scanning parameters were as follows: ratio of repetition time to echo time (TR/TE) (2,000/30 ms), slice thickness (5 mm), pitch (1 mm), field of view (240 × 240 mm), and flip angle (90°). On the structural scan (T1-weighted), the following settings were used: spin-echo sequence, repetition time (TR) = 20 ms, echo time (TE) = 3.5 ms, slice thickness = 1 mm, and field of view (FOV) = 24 × 24 cm.” [excerpted from our previous study (15)].

### Data Preprocessing

Imaging data of rs-fMRI were preprocessed using the DPARSF software (18) in MATLAB (Mathworks). “After signal stabilization, head motion and slice-timing correction were conducted (19, 20). The subjects had a maximal translation ≤ 2 mm in the x, y, or z direction and an angular rotation ≤ 2° on each axis. The functional images were normalized to the standard template in Montreal Neurological Institute (MNI) template and spatially resampled to a voxel size of 3 mm × 3 mm × 3 mm. The head motion parameters obtained by rigid body correction, the white matter signal, and the cerebrospinal fluid signal were removed from the images by linear regression. The signal was bandpass filtered (0.01–0.1 Hz) and linearly detrended to reduce high-frequency physiological noise and low-frequency drift. The global signal removal may introduce artifacts into the data and distort resting-state connectivity patterns. Furthermore, the regression of the global signal may significantly distort results when studying clinical populations. Therefore, the global signal was preserved (21, 22)” [excerpted from our previous study (15)].

### Ventral Somatomotor Network Identification

The toolbox GIFT^1^ was used to pick out VSN as a mask from all participants through the group ICA method. “Three steps from the GIFT toolbox were used as following: data reduction, separation of independent components, and back rebuilding. On the consideration of every component, the voxel-wise one-sample *t*-test set a statistical map and a threshold. Based on Gaussian random field (GRF) theory, *p* < 0.01 represents a significant statistical modification of multiple comparisons. Voxel significance: *p* < 0.01, and cluster significance: *p* < 0.01). Masks were created for the VSN components. Finally, the masks were combined to generate a VSN mask utilized in the following NH analysis.” [excerpted from our previous study (15)].

### Network Homogeneity Analysis

MATLAB was used for NH analysis (14). “For each patient, the correlation coefficients were obtained in a given voxel with all other voxels within the VSN mask. The mean correlation coefficient was defined as the homogeneity of the given voxel, and subsequently changed into *z*-value by using z-transformation to improve the normal distribution as described. The resultant values generated the NH map that finally underwent z-transformation for group comparison” [excerpted from our previous study (15)].

### Statistical Analyses

Demographic information, including sex, age, education degree, and imaging data, were calculated between the patient and control groups. Non-parametric Kruskal-Wallis test was used to compare the distributions between multiple groups because not all samples were in compliance with normal distribution. Categorical data were analyzed with a chi-square test using the IBMSPSS Statistics 22.0 software. Analyses of covariance were executed to compare differences across the three groups on voxel-based VSN maps. Then, *post-hoc t*-tests were executed to compare VSN differences between every two groups. Sex, age, years of education, and head motion were applied as covariates in group comparisons to limit the possible effects of these components. The significance level was set at the corrected *p* < 0.01 for multiple comparisons using the Gaussian Random Field (GRF) theory (GRF-corrected, voxel significance: *p* < 0.001, cluster significance: *p* < 0.01). Correlations between clinical variables were analyzed using partial correlations with head motion as a covariate. Bonferroni correction for multiple comparisons was used in the correlation analysis.

## Results

### Demographics and Clinical Characteristics of Subjects

In this study, 135 TLE patients (52 lTLE patients and 83 rTLE patients) and 68 age- and sex-matched healthy controls were recruited for the study. The demographic and clinical characteristics of the study subjects are provided in Table 1. No significant differences were observed among the three groups regarding age, sex, and years of education. The patient group showed a longer executive control reaction time.

**TABLE 1:** Characteristics of the participants.

Characteristics	NC (*n* = 68)	rTLE (*n* = 83)	lTLE (*n* = 52)
Gender (male/female)	68 (36/32)	83 (43/40)	52 (33/19)
Age, years	26.55 ± 4.90	28.64 ± 8.52	27.74 ± 7.89
Years of education, years	12.32 ± 2.40	11.89 ± 2.68	11.73 ± 2.01
Illness duration, years	–	8.63 ± 7.04	7.98 ± 6.70
ECRT	72.88 ± 36.03	129.22 ± 42.95*	124.07 ± 31.96*
Head motion	0.11 ± 0.03	0.10 ± 0.04	0.09 ± 0.03

A non-parametric statistics (Kruskal-Wallis test) was used for continuous data, and the X^2^ test for categorical data. Compared with normal controls, *P < 0.01. NC, normal controls; RT, reaction time.

### Ventral Somatomotor Network Maps Determined by Group Independent Component Analysis

**FIGURE 1:**
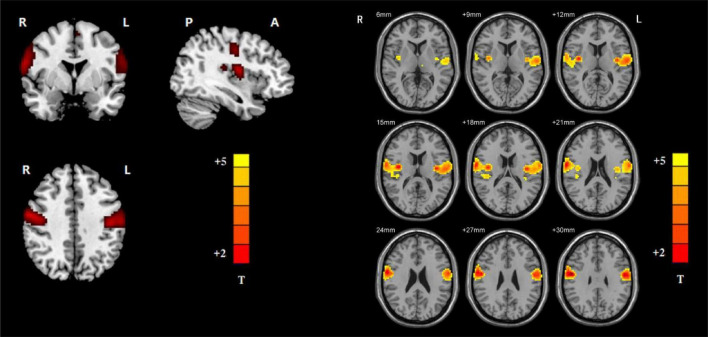
Ventral somatomotor network (left: axial, coronal, and sagittal images of VSN; right: the detailed axial images of VSN. Based on group ICA with a threshold at z ≥ 5).

By employing ICA, the VSN masks were chosen from the control group. The parts involved in the VSN were the bilateral postcentral and Rolandic operculum (Figure 1). The VSN was used as a mask in the following NH analysis.

### Group Differences in Ventral Somatomotor Network Regarding Network Homogeneity

**FIGURE 2:**
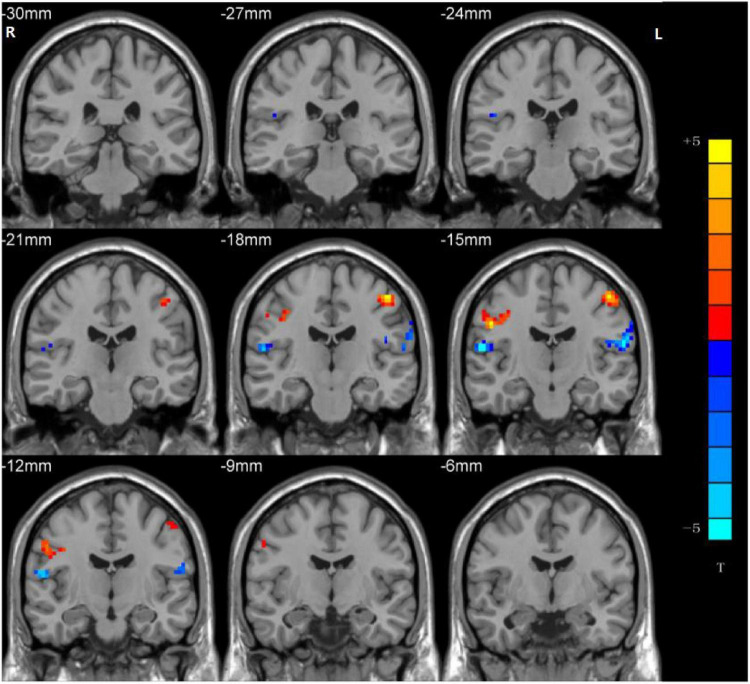
Statistical maps showing NH differences in the bilateral postcentral gyrus, the bilateral Rolandic operculum and the right STG between the rTLE group and control group. (Blue indicates lower NH and the color bar indicates the *t*-values from the two-sample *t*-test).

**FIGURE 3:**
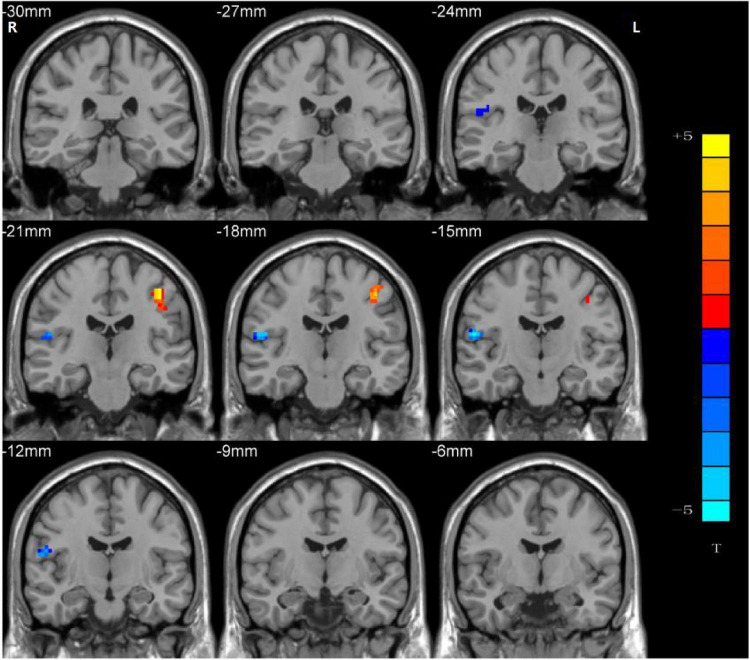
Statistical maps showing NH differences in the left postcentral gyrus and the right Rolandic operculum between the lTLE group and the control group. (Blue indicates lower NH and the color bar indicates the *t*-values from the two-sample *t*-test).

**FIGURE 4:**
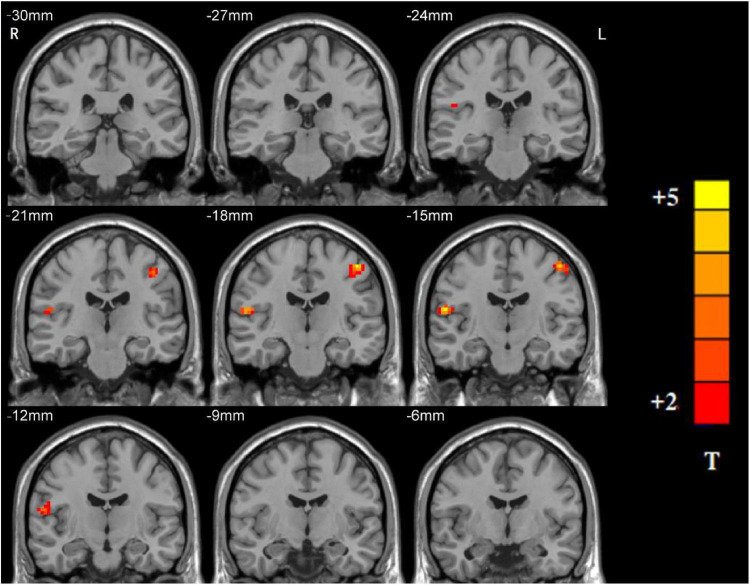
Statistical maps showing NH differences in the right Rolandic operculum and the left postcentral gyrus between the rTLE group and the lTLE group. (Blue indicates lower NH and the color bar indicates the *t*-values from the two-sample *t*-test).

Significant group differences in NH values between the patients (rTLE/lTLE) and the controls within the VSN mask were observed *via* two-sample *t*-tests. Compared to the controls, rTLE patients had significantly higher NH in the bilateral postcentral gyrus, and significantly lower NH in the bilateral Rolandic operculum and right superior temporal gyrus (STG) (Figure 2). The NH values of the left postcentral gyrus were significantly higher in lTLE patients than in the healthy controls, and lTLE patients had lower NH in the right Rolandic operculum (Figure 3). The NH values of the right Rolandic operculum and left postcentral gyrus were significantly higher in rTLE patients than in the lTLE patients (Figure 4).

### Correlation of Network Homogeneity With Clinical Variables

**FIGURE 5:**
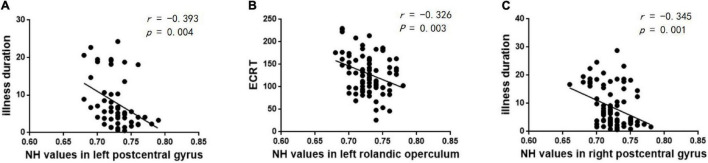
Correlations between abnormal NH and clinical variables. **(A)** Negative correlation between the NH values in the left postcentral gyrus and illness duration in the lTLE group. **(B)** Negative correlation between the NH values in the left Rolandic operculum and ECRT in the rTLE group. **(C)** Negative correlation between the NH values in the right postcentral gyrus and illness duration in the rTLE group.

The correlations between abnormal NH and clinical variables in the patients were examined. The increased NH of the left postcentral gyrus was negatively correlated with the illness duration in the lTLE group (*r* = –0.393, *p* = 0.004) (Figure 5A). The increased NH of the right postcentral gyrus was negatively correlated with the illness duration in the rTLE group (*r* = –0.345, *p* = 0.001) (Figure 5C). And the decreased NH in the left Rolandic operculum was negatively correlated with the ECRT in the rTLE group (*r* = –0.326, *p* = 0.003) (Figure 5B). No other correlations were observed in the participants.

## Discussion

Temporal lobe epilepsy is the most common drug-resistant form of epilepsy in adults (23) and is the most common indication for surgical intervention. However, surgical treatment is invasive and has several undesirable and serious side effects, and not every patient is a candidate for surgical intervention. Given the limited treatment options available for those with drug-resistant TLE, it is crucial to understand the pathophysiologic mechanism of TLE. Because TLE patients usually have motor and sensory impairment (24), we aimed to investigate the abnormal ventral somatomotor network in patients with rTLE/lTLE. In this study, we used an NH method to investigate the VSN region, which is associated with motor and somatosensory function, in patients with TLE (rTLE/lTLE). Compared to healthy controls, rTLE patients had significantly higher NH in the bilateral postcentral gyrus and significantly lower NH in the bilateral Rolandic operculum. Compared to healthy controls, lTLE patients had significantly higher NH in the left postcentral gyrus and lower NH in the right Rolandic operculum. These patients had a longer ECRT. In addition, the altered NH in the postcentral gyrus was negatively correlated with the illness duration, and the decreased NH in the left Rolandic operculum was negatively correlated with the ECRT. We speculated that abnormal NH in the postcentral gyrus and Rolandic operculum play a critical role in the pathophysiology of TLE.

The postcentral gyrus, which is located on the lateral surface of the parietal lobe between the central sulcus and postcentral sulcus and caudal to the central sulcus, corresponds to Brodmann areas 3b, 1, and 2 (25), which contain the primary somatosensory network and are thereby crucial for proprioception and motor control (26). Furthermore, the postcentral gyrus also includes the secondary somatosensory network, which is involved in integrating memories with somatosensory stimuli (27). Because of its unique characteristics, the postcentral gyrus is often affected in psychiatric illness. Li et al. (28) and Kilts et al. (29) found that increased activity of the postcentral gyrus is associated with decreased social anxiety symptoms. Zhuo et al. (30) reported decreased density in the resting-state global functional connectivity of the postcentral gyrus in patients with major depressive disorder. Larabi et al. (31) observed lowered activation of the postcentral gyrus during suppression, which resulted in poorer self-reflectiveness in schizophrenia patients. Jalbrzikowski et al. (32) confirmed that increased postcentral surface area is associated with less severe negative symptoms and better executive cognition in bipolar disorder. With a whole-brain voxel-based unbiased resting-state functional connectivity method, Cheng et al. (33) demonstrated reduced connectivity in the bilateral postcentral gyrus of patients with autism. Song et al. (34) demonstrated that destructive lesions in the postcentral gyrus, as well as changes in its outflow pathways to neighboring motor areas, could lead to epileptic negative myoclonus. These findings could be attributed to the location, structure and function of the postcentral gyrus. As reported by DiGuiseppi et al. (35), axons from the ventral posterolateral nucleus travel from the thalamus through the posterior limb of the internal capsule and terminate in the appropriate region of the postcentral gyrus. The postcentral gyrus has numerous connections with other brain areas, including the insula (36), amygdala (37), limbic system (38), cerebellum (39), and parietal lobe (40). Because of these numerous connections, the postcentral gyrus is able to perform a variety of functions, including those involved in somatic perceptual processes. The postcentral gyrus can also integrate sensory-motor connections associated with poor attentional set shifting and participate in suppression and cognitive insight because emotion-specific representations convey emotions and emotion processing. Our results showed significantly higher NH in the bilateral postcentral gyrus in patients with rTLE and higher NH in the left postcentral gyrus in patients with lTLE, which is consistent with previous studies (41–43). Furthermore, the altered NH values of the left postcentral gyrus were negatively correlated with the duration of the illness in the lTLE group. And similar finding was observed in the rTLE group that altered NH values of the right postcentral gyrus were negatively correlated with the illness duration. We speculate that as rTLE/lTLE progressed, the correlation of the postcentral region with all other regions in the VSN decreased, resulting in functional loss in the postcentral region. We hypothesize that altered NH values in the postcentral gyrus may be responsible for cognitive dysfunctions in TLE patients, including executive cognition (32), attention alertness (44), emotion processing (29), and primary information processing.

The Rolandic operculum is defined as the confluence of the most caudal parts of the pre- and postcentral gyri, is contiguous to the oropharyngeal muscle control area of M1S1 and adjacent to the insula, and corresponds to Brodmann areas 6, 4, and 43 (45, 46). The most well-known function of the Rolandic operculum is its contribution to articulation and tongue movement during speech production, as it includes the ventral portion of the somatotopic tongue and lip representations (47). Furthermore, many other functions of the Rolandic operculum have been discovered in recent years. Fink et al. (48) found that an activated Rolandic operculum supports multimodal input processing from various motor, sensory, and perceptual sources. Ventre-Dominey (49) reported that the Rolandic operculum overlaps with a cortical network that is thought to be associated with self-referential processes that involve self-location in space. Blefari et al. (50) confirmed that the Rolandic operculum is crucial for interoceptive awareness and bodily self-consciousness by processing integrated exteroceptive-interoceptive signals. The findings of Wu et al. (51) suggest that functional impairments of the left Rolandic operculum in patients with schizophrenia are related to delusional thoughts, and patients with schizophrenia have been found to have increased mean connectivity in the left Rolandic operculum (52). Shan et al. (53) discovered that an activated Rolandic operculum is associated with cognitive disabilities in Alzheimer’s disease and mild cognitive impairment. Wang et al. (54) reported that the Rolandic operculum is associated with the neural mechanisms of tic generation. Decades ago, it was discovered that critical electrical discharges in temporal lobe epilepsy always affect extratemporal structures, such as the Rolandic operculum (55). In the present study, lower NH was found in the bilateral Rolandic operculum of patients with rTLE and in the right Rolandic operculum of patients with lTLE. Furthermore, we found that decreased NH in the left Rolandic operculum was negatively correlated with the ECRT between the control group and rTLE group. A longer ECRT indicates inferior executive control performance, and thus, we speculated that abnormal NH in the left Rolandic operculum played a critical role in the executive function of patients with rTLE.

In the present study, we observed that rTLE and lTLE patients had altered NH values in the postcentral gyrus and Rolandic operculum. Because the postcentral gyrus and Rolandic operculum in play critical roles in cognitive function, we speculate that network homogeneity abnormalities caused by temporal lobe epilepsy may account for cognitive dysfunctions in TLE patients. Additionally, this may be the cause of abnormal electrical discharge in patients with TLE. However, our results are consistent with those of some previous studies (56, 57). Li et al. (56) found that increased functional connectivity of the Rolandic operculum was lateralized in patients with rTLE, and Zhou et al. (57) reported that decreased functional connectivity of the postcentral gyrus was lateralized in patients with rTLE. However, we found that the altered NH of the Rolandic operculum and postcentral gyrus were bilateral in patients with rTLE. We speculate that the following factors may account for this phenomenon. (1) There might be a compensatory mechanism that occurs in patients with lTLE/rTLE at rest. As part of the VSN, the postcentral gyrus and the Rolandic operculum share many similarities and overlaps in function; for example, they are both closely related to depression, schizophrenia, epilepsy, and emotion and sensation processing. Bettus et al. (58) reported decreased basal functional connectivity within epileptogenic networks with concurrent contralaterally increased connectivity, which confirmed our suspicions. Campo et al. (59) discovered decreased effective connectivity in the medial temporal lobe of the lesional hemisphere and increased effective connectivity in the medial temporal lobe/inferior frontal cortex of the contralesional hemisphere. Similar to these findings regarding functional connectivity in patients with epilepsy, our results demonstrated increased NH values in the ipsilesional postcentral gyrus and decreased NH values in the contralesional Rolandic operculum. (2) Because TLE is a chronic disorder, epileptic discharges propagating into the contralateral gyrus and recurrent epileptic activities might be responsible for the contralateral structural and functional decline as TLE progressed. Jokeit et al. (60) confirmed the TLE patients had altered glucose metabolism in the contralateral hippocampus, which was related to seizure frequency. Additionally, the duration of TLE may have a greater impact on the contralateral measure of hippocampal metabolism than the ipsilateral measure. Following a multiple regression analysis, there was a subsequent decrease in the hippocampal volume contralateral to the primary temporal seizure focus (61, 62). We believe that similar processing occurs in the postcentral gyrus and Rolandic operculum, which is strongly supported by the results of our study.

The STG plays a key role in language function (63), and study showed that the activity of STG was strongly associated with the listeners’ subjective experience (64). A fMRI study enrolled 43 TLE patients with language-impaired and 42 TLE patients with non-language-impaired confirmed that activations within the STG was the most predictive of language impairment (65). A Chinese tasks based fMRI study showed that the main effect region of auditory naming and picture naming tasks was in right STG, and the main effect region of semantic fluency task was in left STG (66). Meanwhile, to protect the language function in the postoperative patients, the task-state fMRI and intraoperative electroencephalography were suggested to be used to develop a personalized surgical plan for epilepsy treatment (66). Moreover, the dysfunction in the STG was related to the cognitive dysfunction in epilepsy patients. A rs-fMRI study found that decreased functional connectivity of the left precentral gyrus with the bilateral STG has a significant effect on intelligence in children and adolescents with focal epilepsy (67). The right STG was found blunted neural response to emotional faces in pediatric epilepsy, which was regarded as a marker of risk for social cognitive deficits (68). In our study, lower NH in the right STG was found in the rTLE, and no significant correlation was found between the NH and clinical variables including the ECRT. Based on these prior studies, we speculate that the decreased NH was associated with the language function in TLE, though relevant language task hadn’t perform in our study.

There were several limitations in the study: (1) Physiological noise cannot be completely removed. (2) We focused only on abnormalities of the VSN in patients with TLE, and although illuminating the pathophysiological contribution of the VSN is critical, other significant brain networks might have been neglected. (3) Antiepileptic drugs may also have effects on functional networks in epilepsy, and we did not account for this confounding factor.

## Conclusion

In conclusion, we used the NH method to analyze resting-state fMRI data in patients with TLE, and abnormal NH values in the VSN were confirmed. The importance of NH alterations in the VSN implies the importance of the postcentral gyrus, STG and Rolandic operculum in the progression of TLE and provides new insights into the pathophysiological mechanism of TLE.

## Data Availability Statement

The raw data supporting the conclusions of this article will be made available by the authors, without undue reservation.

## Ethics Statement

The studies involving human participants were reviewed and approved by the Medical Ethics Committee of the Tianyou Hospital Affiliated to Wuhan University of Science and Technology. The patients/participants provided their written informed consent to participate in this study. Written informed consent was obtained from the individual(s) for the publication of any potentially identifiable images or data included in this article.

## Author Contributions

DL and LM: writing the article. PX, HR, and RL: collection and assembly of data. LZ and YG: research concept and design, data analysis, and interpretation. All authors contributed to the article and approved the submitted version.

## Conflict of Interest

The authors declare that the research was conducted in the absence of any commercial or financial relationships that could be construed as a potential conflict of interest.

## Publisher’s Note

All claims expressed in this article are solely those of the authors and do not necessarily represent those of their affiliated organizations, or those of the publisher, the editors and the reviewers. Any product that may be evaluated in this article, or claim that may be made by its manufacturer, is not guaranteed or endorsed by the publisher.

## Footnotes

1
http://mialab.mrn.org/software/#gica
